# Study of the Effectiveness of Skin Restoration Using a Biopolymer Hydrogel Scaffold with Encapsulated Mesenchymal Stem Cells

**DOI:** 10.3390/ijms26167840

**Published:** 2025-08-14

**Authors:** Marfa N. Egorikhina, Lidia B. Timofeeva, Yulia P. Rubtsova, Ekaterina A. Farafontova, Dariya D. Linkova, Irina N. Charykova, Maksim G. Ryabkov, Anna A. Ezhevskaya, Ekaterina A. Levicheva, Diana Ya. Aleynik

**Affiliations:** Federal State Budgetary Educational Institution of Higher Education, Privolzhsky Research Medical University of the Ministry of Health of the Russian Federation, 603005 Nizhny Novgorod, Russia; bioli@mail.ru (L.B.T.); ekaterina_farafontova@mail.ru (E.A.F.); linckovadaria@yandex.ru (D.D.L.); irina-ch0709@yandex.ru (I.N.C.); maxim-ryabkov@yandex.ru (M.G.R.); annaezhe@yandex.ru (A.A.E.); kate.lekat@yandex.ru (E.A.L.); daleynik@yandex.ru (D.Y.A.)

**Keywords:** scaffold, mesenchymal stem cells, tissue-engineered constructs, preclinical studies, pigs, biocompatibility, regeneration, biopolymers

## Abstract

Improving the restoration of skin defects of various etiologies continues to be an important medical challenge globally. This primarily applies to the treatment of chronic wounds and major burns, which create particularly complex and socially significant problems for surgery. In recent decades the progress in these fields has largely been associated with techniques for regenerative medicine, specifically, techniques based on the use of tissue-engineered constructs. Before their use in clinical practice, all such newly developed constructs require preclinical studies to confirm their safety and effectiveness in animal models. This paper presents the results of preclinical studies of the effectiveness of restoration of full-layer degloving wounds in pigs using grafts of either an original biopolymer hydrogel scaffold or a skin equivalent based on it, but seeded with autologous skin cells (ASCs). It is demonstrated that the scaffold itself integrates into the wound bed tissues, facilitating cell recruitment and the accumulation and early maturation of granulation tissue. Then, at later stages of regeneration, the scaffold accelerates the maturation of connective tissue and promotes the formation of tissues similar to those of healthy skin in terms of thickness and structure. Owing to the ASCs present in it, the skin equivalent demonstrates greater effectiveness than the scaffold alone, in particular, due to overall faster remodeling of the graft connective tissue. Therefore, the scaffold we have developed and the skin equivalent based on it have much potential as products for the repair of skin wounds.

## 1. Introduction

Effective and high-quality restoration of skin, following wound damage, has been a pressing issue from early recorded history and is still relevant in the 21st century. Globally, this issue can potentially be solved by using techniques of regenerative medicine, namely, the development of modern tissue-engineered constructs or skin equivalents (SEs). It is generally accepted that the first SE was obtained in vitro in 1976 by E. Freeman et al., who successfully cultivated human skin explants on pig skin collagen from the dermal layer [1]. High-potential multilayer SEs became available 30 years later: epidermal SEs (such as EpiSkin^®^, EpiDerm^®^, SkinEthic^®^), and two-layer constructs modeling both the epidermal and dermal layers (Epiderm FT^®^, StrataTest^®^) [2]. Although the bilayer equivalents were intended to imitate the skin structure more effectively, they found no use in everyday clinical practice. However, such equivalents did become a useful tool for screening and testing the safety of various pharmaceutical and cosmetic products [3,4].

In modern clinical practice, various applied tissue engineering products are referred to as “skin equivalents”. These include cell-free constructs and mono-cell constructs as well as more complex constructs formed from several types of cells, often arranged in layers (Table 1).

Thus, modern skin equivalents are a fairly diverse group of complex constructs, including both cell-free and biologically active cellular products of tissue engineering aimed at stimulating regeneration in regions where the skin has suffered wounding.

The clinical use of SEs can be divided into two stages: the first being the application of a skin substitute matrix to the wound, and the second, the transplantation of an autograft. The gold standard treatment of burns and wounds provides for necrectomy followed by autodermoplasty [14,15]. For these purposes, a split thickness skin graft (STSG) [16] or a full thickness skin graft (FTSG) [17] can be used. STSGs contain both the epidermis and the superficial layer of the dermis, whereas FTSGs provide both epidermis and a full-thickness dermal layer [18]. In light of the range of clinical experience, various types are used during preclinical studies on animals.

B.L. Dearman et al. (2023) in their experiments with a pig model used a two-stage procedure to repair wounds. The investigation was conducted on three large white Landrace pigs. After each of the three pigs was inflicted with four wounds of 8 × 8 cm down to the subcutaneous fat tissue, a biodegradable temporary matrix—NovoSorb™ (BTM)—was implanted into each wound for 28 days. Four pieces of excised skin were processed to isolate autologous ‘donor’ cells—pig fibroblasts (pFbs) and pig keratinocytes (pKs)—and these were then cultured. The resulting cultured cells were used to form three types of SEs: with collagen and glycosaminoglycan (C-GAG); with biodegradable polyurethane foam (PUR); and with a hybrid combination of the two (PUR/C-GAG). After 28 days, the BTMs of each of the three pigs were replaced with one of the three types of SE or with an STSG (perforation ratio: 1:3), which was used as a control. After 16 weeks, in general, all three SEs facilitated wound healing and skin repair due to vascularization and epithelialization [15].

However, such a two-stage treatment has the following disadvantages: possible scar formation and an increased risk of chronic wounds and infection of the donor site. In clinical practice, skin from donor sites for autodermoplasty has to be taken several times when treating major wounds and burns. As a result, a two-stage procedure requires multiple interventions, and the duration of damaged tissue restoration and complete epithelialization of the wound increases significantly [19,20]. Therefore, scientists are now exploring options for a one-stage procedure that would reduce the number of surgical interventions, avoid the disadvantages of two-stage treatment, and reduce the overall financial costs.

To eliminate some of the disadvantages of the two-stage procedure for skin restoration, new types of SEs are being actively developed in order to avoid the need to use autografts. Such SEs often mimic the extracellular matrix to ensure wound coverage, prevent fluid loss, and provide for cellular infiltration. In use, such SEs can be subject to progressive remodeling, and this can contribute to functional tissue restoration [21]. The first attempts at independent SE application were made in 2007 by F.M. Wood et al. The study investigated the effects of a one-step application of an uncultured autologous cell suspension, isolated using the ReCell device in combination with Integra^®^, for skin regeneration. The experiment was conducted on two female Yorkshire pigs, in which 10 full-thickness wounds of 5 × 5 cm were used as the model; the animals were observed for 35 days. The results of this pilot study showed that the combination of Integra^®^ with the autologous cells facilitates one-step reconstruction after a full-thickness skin wound in experimental animals [20].

In another study [22], full-thickness thermal burns were formed on the backs of eight Gottingen minipigs. Two days after injury, the wounds were fascially excised, and the animals were randomized to be grafted either with SEs made using Integra^®^ seeded with non-cultured ADRCs (adipose-derived regenerative cells—autologous cells isolated from the inguinal area of the pigs) or Integra^®^ with control medium. Histological analysis showed accelerated maturation of the wound bed tissue in the experimental wounds (Integra^®^ with ADRCs) compared to the controls. The density of blood vessels in the experimental wound beds was 50–69.6% higher compared to that in the controls. On Day 21, an increase in the lumen area of the blood vessels was also evident in the experimental wounds, and the vessels were more mature. The authors concluded that seeding ADRCs onto the SE enhances wound angiogenesis, vascular maturation, and matrix remodeling [22].

In another study, the authors attempted to monitor the vascularization of a Lando^®^ dermal scaffold in full-thickness pig skin wound models [23]. Eight Tibetan pigs were used as experimental animals; each had six 5 × 5 cm full-thickness wounds formed on the back down to the muscular fascia (three control wounds, three experimental wounds). The overall condition of the wounds was observed on Days 3, 7, 14, and 21 after injury. The results showed that the experimental wounds (Lando) were drier with a lower infection rate. In the experimental wounds, granulation tissue had formed more intensively compared to the control wounds and was smoother, without evident edema or redness of the skin around the wound. In the experimental wounds, fibroblasts appeared earlier, capillaries grew mainly parallel to the wound surface (resembling normal skin), and the collagen fibers were thicker and more evenly distributed compared to the control group. The results of immunohistochemical studies (CD31 and a-SMA) recorded proliferative activity of endothelial cells on Day 14 after injury, while a trend to maturation could be noted on Day 21 after injury. The data correlated with VEGF expression. The authors concluded that the use of a tissue-engineered dermal scaffold material provides good results in a pig wound model with a full-thickness defect [23].

In a study by Damaraju S.M. et al. (2022), the feasibility of a one-stage procedure was assessed by combining a bioengineered construct based on collagen-chondroitin-6-sulphate (DS1) or decellularized fetal bovine skin tissues (DS2) with an autologous skin cell (ASC) suspension. The experiment was conducted with eleven female Yorkshire domestic pigs. Each animal was used to model 12 wounds (six different treatment options) of 4 × 4 cm. The maximum observation period for the animals was 42 days. Wounds in the control group (no additional treatment) or with ASCs only showed active wound healing with the formation of fibrous tissues. The combination of ASCs with DS1 and DS2 facilitated the reduction in wound contracture, which was further confirmed by a significant decrease in the myofibroblast population in the experimental wounds compared to the controls and to the ASC-only wounds. Furthermore, the combined use of ASCs with DS1 and DS2 resulted in a decrease in the overall inflammatory response compared to treatment with DS1 or DS2 alone. Throughout the study, control wounds and ASC-only wounds showed higher mature collagen values than in the groups having DS1 or DS2 combined with ASCs. The combination of ASCs with DS1 or DS2 resulted in a significant increase in the percentage of secondary wound epithelialization after 14 days, from 15 to 71% (DS1 compared to DS1 + ASCs) or from 28 to 77% (DS2 compared to DS2 + ASCs). These results confirmed the effectiveness of a one-step approach with SE in clinical practice [21].

Clinical and experimental experience, therefore, demonstrates the relevance of developing new SEs that allow one-step procedures for the restoration of skin wound defects. The current approach is therefore aimed at solving specific clinical problems, such as minimizing the need for restoration of donor wounds, issues of the lack of donor resources—autografts—and reduction in the required number of surgical interventions. Any transition from a two-stage to a one-stage procedure should also increase the effectiveness of treatment by activating regeneration and reducing the number of undesirable consequences, such as the formation of chronic wounds and contractures.

Our research team has developed a skin equivalent utilizing a biopolymer hydrogel scaffold with encapsulated mesenchymal stem cells. Comprehensive preclinical in vitro studies have shown that the scaffold can act as an artificial cellular niche for MSCs, facilitating dynamic reciprocal interactions [24]. In vivo studies have shown that implantation into animal (rat) tissues leads to complete replacement of the acellular scaffold and SE with connective tissue components [25]. The absence of an acute inflammatory response to the implantation of the scaffold and SE was confirmed. The observed processes of cell recruitment to the scaffold from surrounding tissues, active formation of collagen fibers, and absence of acute inflammation indicated the occurrence of the regeneration process in the implantation area. The obtained data confirming the high biocompatibility of SE allowed us to proceed to the next stage of research aimed at evaluating the effectiveness of SE in skin restoration processes.

Aim of our current study: to assess the effectiveness of pig skin restoration in a full-layer degloving wound model in the presence of an original skin equivalent based on a biopolymer hydrogel scaffold with encapsulated allogeneic mesenchymal cells derived from adipose tissue.

## 2. Results and Discussion

### 2.1. Characteristics of the Condition of the Wounds on Day 7 of the Study

Assessment of the condition of the recovering wounds (six wounds) from the three experimental animals revealed that the course of regeneration in wounds of the same type but in different animals had individual peculiarities. Immediately after wound formation, the experimental animals underwent profuse capillary bleeding in the wound area. To stop this capillary bleeding, each wound had been tamponaded for 2 min using a sterile gauze swab soaked in adrenaline solution (1 mg/mL, Moscow Endocrine Plant FSUE, Moscow, Russia). Only after this were the wounds covered with the appropriate mSc or mSE grafts, depending on their type (Con, mSc, mSE), and protective dressings. However, despite the measures taken to stop capillary bleeding, in some cases it had resumed after closure of the wound. In such wounds, the formation of hemorrhages of varying severity was noted, resulting in certain nuances of regeneration and reflected in the overall histological state of the wounds. It should be noted that the blood clots formed after such hemorrhaging, in fact, create a natural scaffold that facilitates granulation tissue formation and tissue regeneration in the wound area.

**Figure 1:**
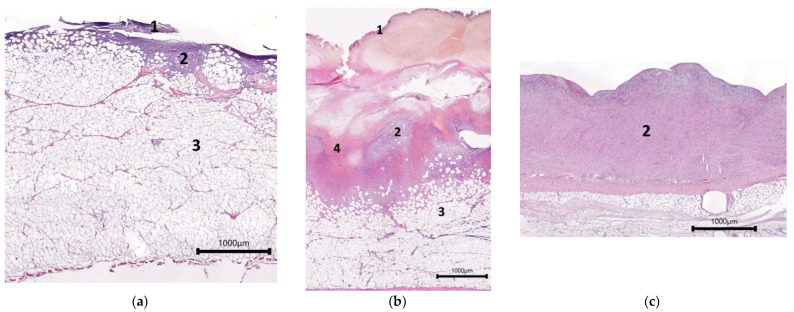

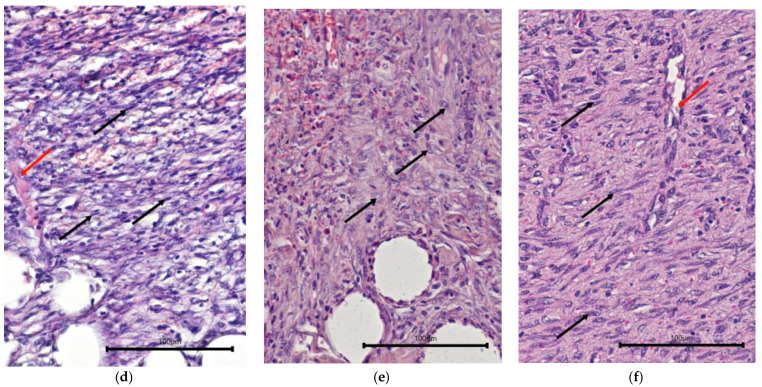
Histological view showing the condition of the central parts of control wound defects on Day 7—hematoxylin and eosin staining: (**a**–**c**) overall views; (**d**–**f**) structure of granulation tissue. Note: 1—leukocyte–necrotic scab; 2—granulation tissue; 3—adipose tissue; 4—hemorrhagic exudate; black arrow—fibroblasts; red arrow—a vertical blood vessel.

For instance, when assessing the control wounds (Con) on Day 7, it was noted that most of the area of the wound defect was covered with a leukocyte–necrotic layer (Figure 1a,b). At the same time, a thin layer of fibrinous and hemorrhagic exudate was seen on the surface of some wounds (Figure 1c). The extent of filling of the wound crater with granulation tissue varied between wounds. In some cases, a thin layer of granulation tissue not filling the wound crater was observed at the bottom of the wound (Figure 1a). In such cases, there was no fibrinous exudate. In other cases, the crater of the wound defect was filled with granulation tissue, located either in a mosaic pattern as foci or completely lining the bottom of the wound defect (Figure 1b,c). Presumably, such uneven formation of granulation tissue in different wounds was associated with the secondary hemorrhages described above. In all cases, the granulation tissue was characterized by weak infiltration with neutrophils, lymphocytes, giant multinucleated cells, and single eosinophils. In all the control wounds, fibroblasts were seen. However, their activity varied. For instance, in wounds with a thin layer of granulation tissue, inactive spindle-shaped fibroblasts with dark oval or rod-shaped nuclei predominated (Figure 1d). There were single hemocapillaries with proliferating endothelium in such wounds. In recovering wounds with more granulation tissue, the fibroblasts showed different degrees of synthetic activity, as evidenced by their morphology. For example, in some cases, fibroblasts with large, light nuclei and several nucleoli were seen. Here, the intercellular matrix had a homogeneous structure and was stained with oxyphilic dye, which indicated the accumulation of collagen (Figure 1e). In other cases, two layers of granulation tissue could be distinguished: a superficial layer with a predominance of amorphous components in the extracellular matrix and a large number of fibroblasts, and a deep layer in which fibroblasts were horizontally oriented and had dark rod-shaped or light oval nuclei, which was an indication of different degrees of tissue maturity. In the latter case, a layer with vertically located vessels could also be seen in the granulation tissue (Figure 1f). In some areas, vascular loops had formed.

**Figure 2:**
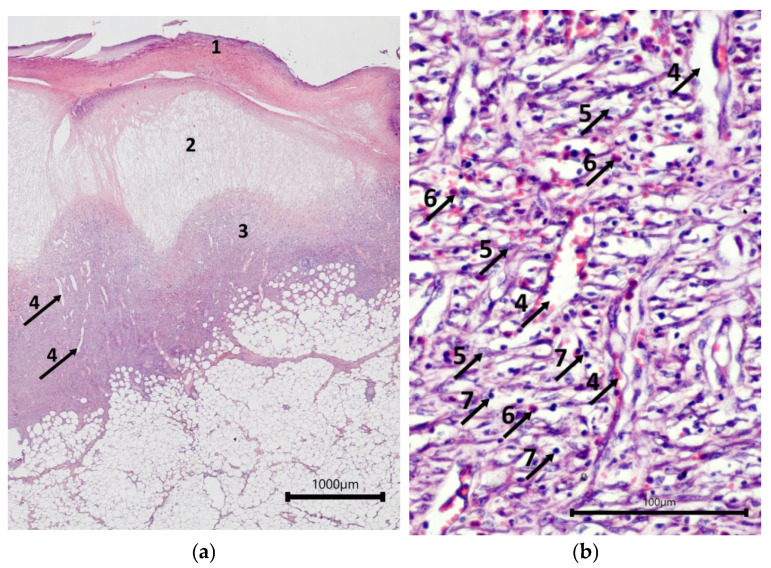
Histological view of the condition of wound defects with model cell-free scaffold on Day 7—showing central part of the wound, hematoxylin and eosin staining. (**a**) Overall view; (**b**) structure of granulation tissue. Note: 1—leukocyte–necrotic layer; 2—fibrin matrix; 3—granulation tissue; 4—vertical blood vessels; 5—fibroblasts; 6—eosinophils; 7—lymphocytes.

Assessment of wound defects with implanted cell-free scaffolds (mScs) showed that their surfaces were covered with a leukocyte–necrotic layer that contained fragments of the scaffold. A layer of fibrinous exudate was located under it; this exudate acted as a matrix for the fibroblasts and endothelial cells that were forming the underlying granulation tissue lining the bottom of the wound crater (Figure 2a). A typical situation for granulation tissue is the presence of a layer with vertical vessels (Figure 2b), but in most cases, no such layers of formed vascular loops were seen. However, there were patches in some wounds with such loops. The granulation tissue contained active fibroblasts with large, light-colored nuclei with one or two nucleoli, synthesizing components of the intercellular matrix. Leukocyte infiltration of varying intensity, from moderate to weak, with a predominance of eosinophils, persisted in the granulation tissue.

**Figure 3:**
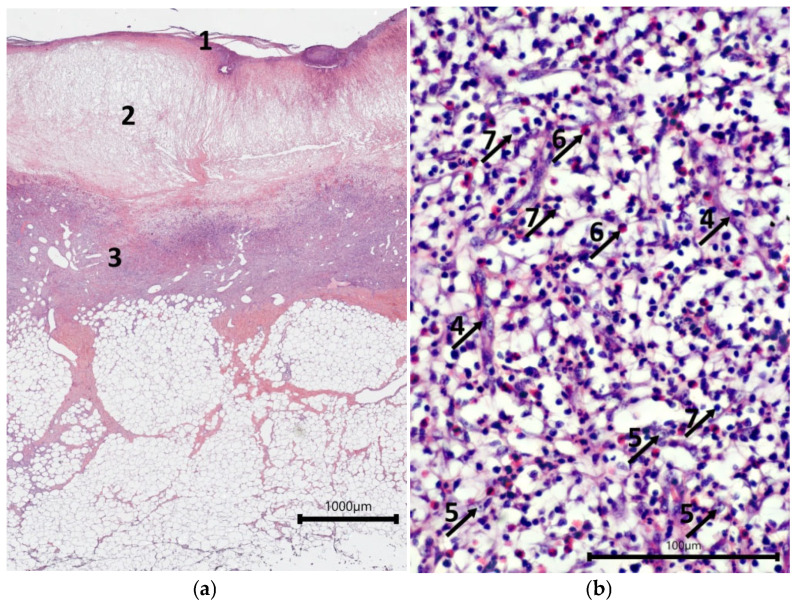
Histological view of the condition of wound defects with mSEs on Day 7—central part of the wound, hematoxylin and eosin staining: (**a**) overall view; (**b**) structure of granulation tissue. Note: 1—leukocyte–necrotic layer; 2—fibrin matrix; 3—granulation tissue; 4—vertical blood vessels; 5—fibroblasts; 6—eosinophils; 7—lymphocytes.

The surfaces of the wound defects with mSEs each had a layer of leukocyte–necrotic scab, consisting of degeneratively changed and destroyed cellular elements, under which a relatively thin layer of fibrinous exudate was seen (Figure 3a). Most of the wound crater was filled with granulation tissue. In some cases, fragments of mSE were found in the granulation tissue. Some mSE was also visualized on the wound surface as part of the leukocyte–necrotic scab. A distinctive feature of the granulation tissue was the extensive leukocyte infiltration. Eosinophils were the predominant type of leukocytes in the infiltrate (Figure 3b). The relative numbers of lymphocytes and neutrophils in the wounds varied. A layer of formed vertical vessels was seen in the granulation tissue. It should be noted that vascular loop formation was not registered. Granulation tissue was characterized by the presence of active fibroblasts.

A common characteristic feature for the animals on Day 7 of the study was a more intense formation of granulation tissue in the wound crater under the mScs and mSEs, if these were used. It should be noted that the use of mScs accelerated the process of granulation tissue formation compared to wounds with mSE grafts, as confirmed by the areas with formed vascular loops on the wound surface.

**Figure 4:**
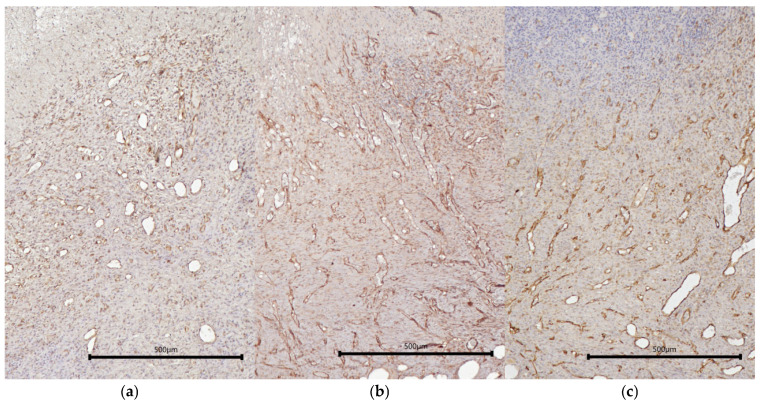
Vessels in granulation tissue. (**a**) Con, (**b**) mSc, (**c**) mSE; CD31 immunohistochemical staining.

**Figure 5:**
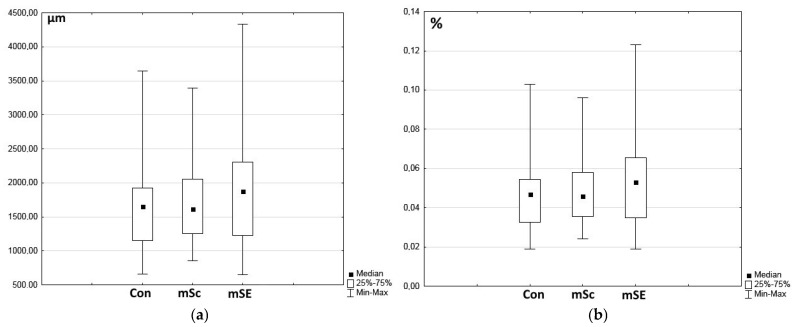
Morphometric examination of vessels. (**a**) Total area of vessels in fields of view of granulation tissue; (**b**) percentage of the vessel area from the total area of fields of view (10 fields of view were analyzed for each wound, so *n* = 60 for each type of wound).

Vessels of different diameters could be clearly visualized in the area of granulation tissue in all types of wounds, being immunohistochemically stained using CD31 (Figure 4). There were no statistically significant differences found between wound defects during morphometric assessment of either the total area of vessels within the granulation tissue in the fields of view or of the percentage of the area of vessels from the total area of the field of view. However, in the mSE-treated wounds, a tendency to an increase was noted (Figure 5).

**Figure 6:**
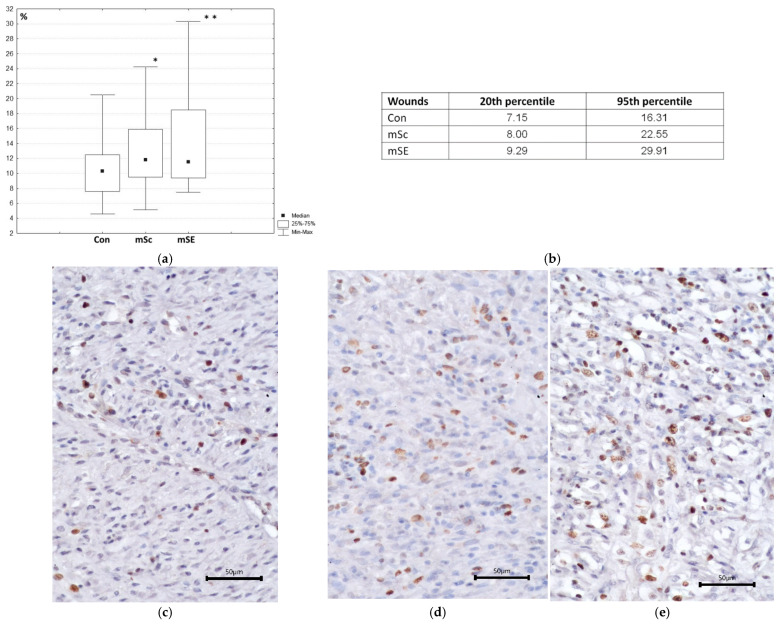
Proliferative activity of cells. (**a**) Percentage of cells expressing Ki-67 relative to the total number of cells in the fields of view (10 fields of view were analyzed from each wound defect, so *n* = 60 for each wound type); (**b**) analysis of data distribution between the 20th and 95th percentiles; (**c**–**e**) proliferating cells in granulation tissue, immunohistochemical staining—Ki-67; (**c**) Con; (**d**) mSc; (**e**) mSE. Note: * *p* ˂ 0.05; ** *p* ˂ 0.001 in comparison with Con, using the Wilcoxon test.

More active regeneration, where mScs and mSEs were used, was confirmed by the results of morphometric examination. For instance, the percentage of cells expressing Ki-67, a marker of proliferating cells, relative to the total number of cells in the field of view, was statistically significantly higher in wounds with mSc and mSE grafts compared to the control wounds (Figure 6a). However, the assessed indicator did not statistically significantly differ between wounds with mSc or mSE grafts. Sample analysis showed that 75% of the values for the assessed wound defect index with mSEs were within the range from 9.29 to 29.91%, whereas in the control wounds, they were within the range from 7.15 to 16.31% (Figure 6b), indicating that the proliferative activity of cells in wounds with mSEs was more pronounced. A total of 75% of the values for the assessed wound defect index with mScs were within the range from 8.00 to 22.55% and had an intermediate value between the control wounds and wounds with mSEs. Thus, both the mSc and mSE grafts enabled cells to maintain pronounced proliferative activity. This could be due to the biological activity and structure of these scaffolds. In the case of mSEs, the effect was more pronounced, probably due to the pASCs in the mSE, as these secrete growth factors and interleukins capable of impacting the proliferative activity of cells.

**Figure 7:**
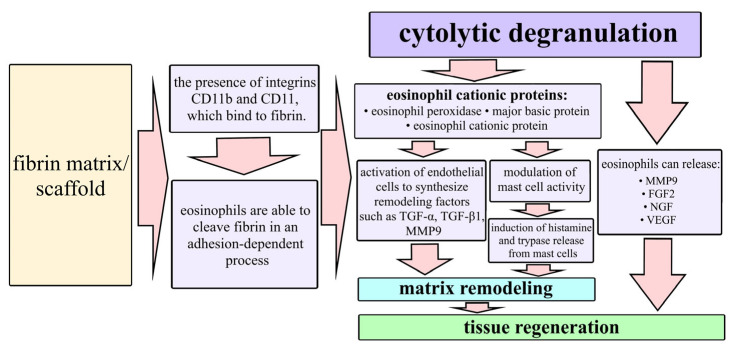
Interaction of eosinophils with the fibrin matrix and participation in regeneration.

In wounds with mSc and mSE grafts, abundant infiltration of granulation tissue with eosinophils (Figure 5b and Figure 6b) was noted. Eosinophils express a unique complex of cell surface receptors related to adhesion, homing, and migration in tissues [26,27]. It is known that the eosinophil membrane contains integrins CD11b and CD11, which bind to fibrin [26,28,29,30,31] and that eosinophils can cleave fibrin in an adhesion-dependent manner [32]. M.E. Coden et al. also showed that fibrin is a specific trigger for the cytolytic mode of eosinophil degranulation. As fibrin is the main structure-forming protein of the scaffolds of the implantable mSc and mSE constructs, presumably, this fibrin “attracts” eosinophils from the surrounding tissues and causes their active recruitment into the structure of the constructs. Relatively recently, eosinophils have begun to be considered as participants in regeneration. During cytolytic degranulation, they release cationic proteins [33,34,35] that can activate epithelial cells to synthesize remodeling factors such as TGF-*α* and TGF-*β* 1 [33] as well as modulating mast cell activity [36,37]. Thus, eosinophils release a number of proteins that are directly involved in tissue regeneration processes [27,38] (Figure 7).

**Figure 8:**
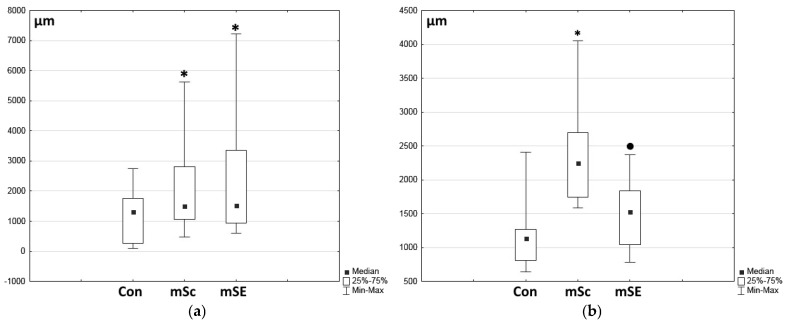
Morphometric assessment of marginal epithelialization and granulation tissue thickness in experimental animal wounds. (**a**) Thickness of granulation tissue filling the wound crater (10 measurements were taken for each wound defect, so *n* = 60 for each wound type), (**b**) length of marginal epithelialization in wound defects (*n* = 12 for each wound type, measurements were taken at the right and left edges of each wound defect sample). Note: * *p* ˂ 0.01 in comparison with Con; ● *p* ˂ 0.01 in comparison with mSe, using Wilcoxon test.

During morphometric assessment of the granulation tissue thickness, it was shown to be minimal in the control wounds, while it was statistically significantly greater in the experimental wounds (Figure 8a). However, no statistically significant differences were seen for this indicator between the mSc and mSE grafted wounds. The extent of marginal epithelialization in the wound defects was also assessed, demonstrating that the marginal epithelialization process in the control wounds on Day 7 was slower than in the experimental wounds (Figure 8b). Marginal epithelialization was most active in wounds with mScs. In the mSE-treated wounds, the marginal epithelialization process was slower compared to that with mScs.

**Figure 9:**
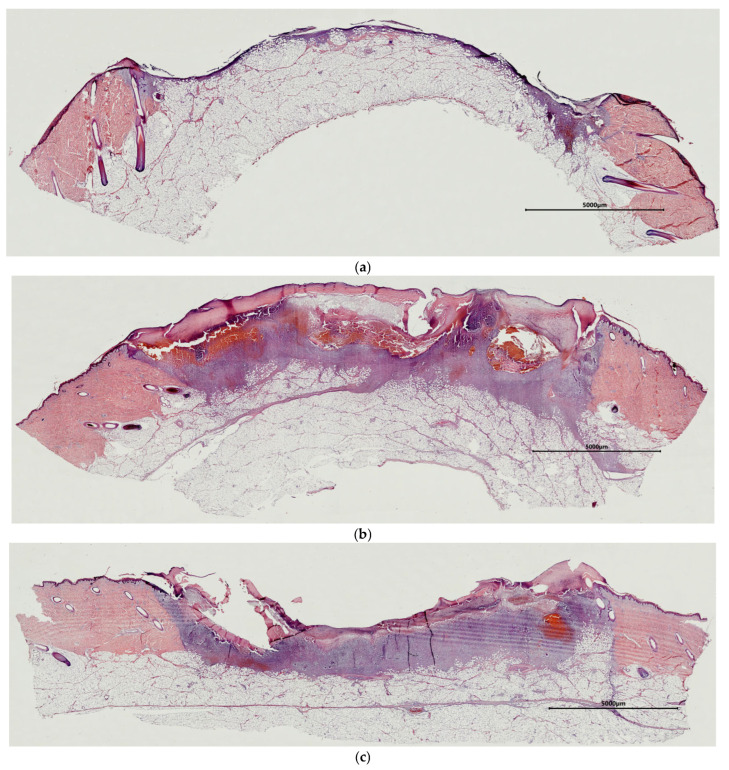
Example of the general appearance of wound defects in one animal on Day 7. (**a**) Con; (**b**) mSc; (**c**) mSE; hematoxylin and eosin staining.

The identified differences are well visualized when comparing wound defects in any individual animal. As an example, an overall view of wound defects from one of the experimental animals is provided, allowing comparison of the differing aspects of regeneration occurring in the control and experimental wounds (Figure 9).

**Figure 10:**
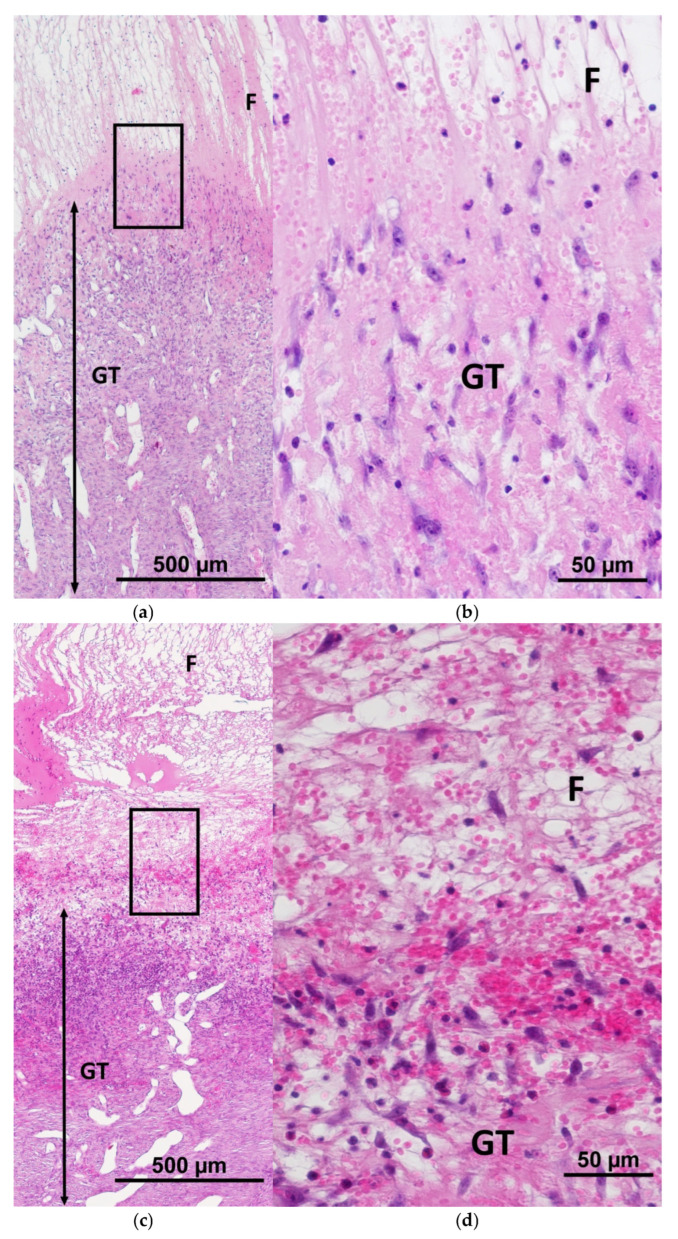

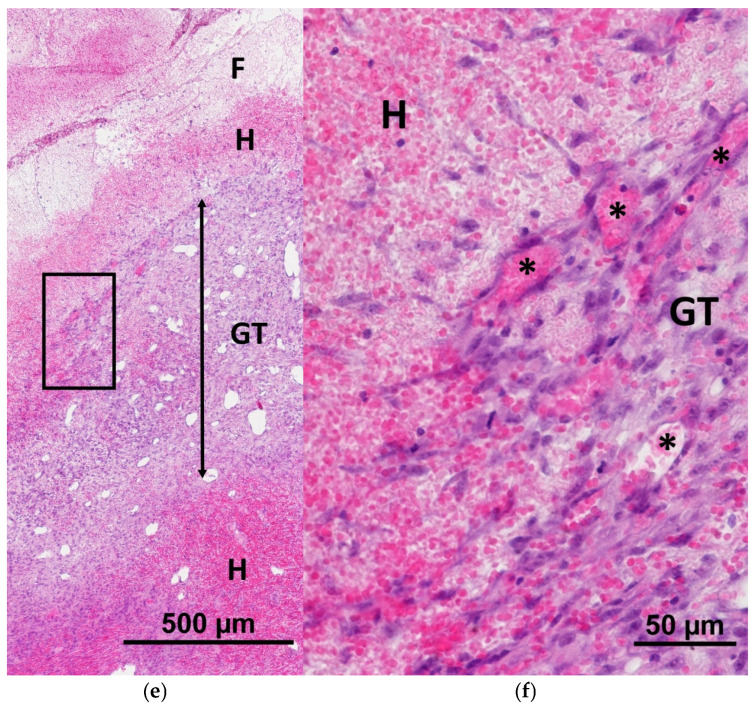
Migration of fibroblast-like cells from granulation tissue into implanted constructs. (**a**,**b**) mScs; (**c**,**d**) mSEs; (**e**,**f**) Con; (**a**,**c**) overall view of the border area of granulation tissue and the implanted constructs in the experimental wounds; (**e**) overall view of the border area of granulation tissue and the hemorrhage area in the control wounds; (**b**,**d**,**f**) cell orientation clearly indicates the direction of migration of fibroblast-like cells. Note: F—fibrin matrix; GT—granulation tissue; H—hemorrhage; *—blood vessels.

One of the critical indicators that ensures successful regeneration is the integration of the implanted constructs into the skin tissues, as evidenced by the colonization of the scaffolds with cells recruited from the surrounding tissues [39,40,41]. Histological assessment on Day 7 showed that mSc and mSE structures could be quite easily visualized in the wounds, as they had both a significantly lower density and a “mesh” structure (Figure 2a and Figure 3a), providing more porosity than the underlying granulation tissue. At higher magnification, it was possible to see the infiltration of both the mSc and mSE scaffolds with leukocyte cells. Active migration of cells with fibroblast-like morphology towards the constructs (upwards) from the underlying granulation tissue was observed (Figure 10a–d). The direction of migration is indicated by the vertical orientation of these cells. In some control wounds where hemorrhaging was observed, migration of fibroblast-like cells into the overlying layers of the grafts was also recorded (Figure 10e,f). A fibrin matrix had formed during hemorrhaging, and this promoted regeneration, acting as a natural scaffold, providing conditions for the recruitment of cells from the underlying granulation tissue.

Summarizing the obtained data, one can conclude that regeneration in the experimental wounds was much more active on Day 7 than the processes in the control wounds. It should be noted that the activity of the regeneration process at this stage of the study was greater in the wound defects treated with mScs, as evidenced by such indicators as the maturity of the granulation tissue and the extent of marginal epithelialization. With the mSE regeneration being at an earlier stage compared to wounds with mSc constructs, the former demonstrated a higher intensity of proliferative activity of cells in the granulation tissue.

### 2.2. Characteristics of the Wound Defect Condition on Day 42 of the Study

**Figure 11:**
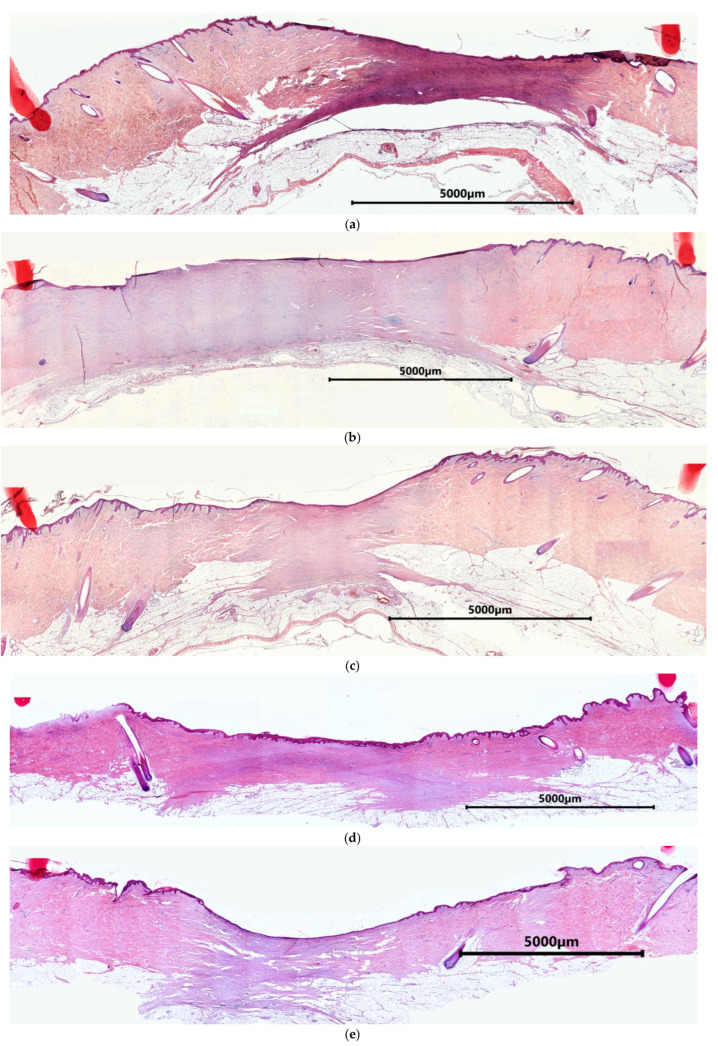

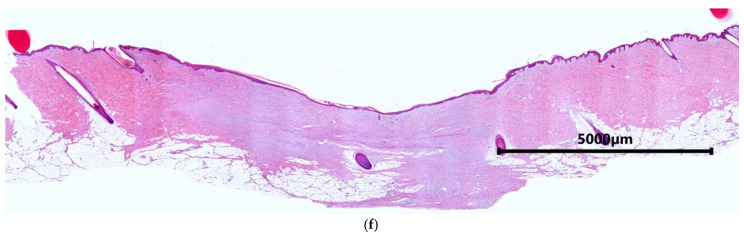
Examples of overall views of wound defects on Day 42 (wound defects of two animals): (**a**–**c**) animal No. 1, (**d**–**f**) animal No. 2; (**a**,**d**) Con, (**b**,**e**) mSc, (**c**,**f**) mSE; hemotoxylin and eosin staining.

By Day 42, the surfaces of all wounds were completely epithelialized. The wound border could not be seen microscopically because the skin structure was completely restored in the peripheral area of the wound crater. Papillary and reticular layers of the dermis were clearly visible under the epidermis in this area, and hair follicles and glands were available (Figure 11). On Day 42, three areas could be distinguished in all types of experimental wounds: (1) the restored dermis, (2) the remodeling area characterized by multidirectional bundles of collagen fibers and active fibroblasts, and (3) the granulation tissue area. Visually, the experimental wound defects typically had a more uniform thickness, and the remodeling area was more extensive than in the control wound defects (Figure 11).

**Figure 12:**
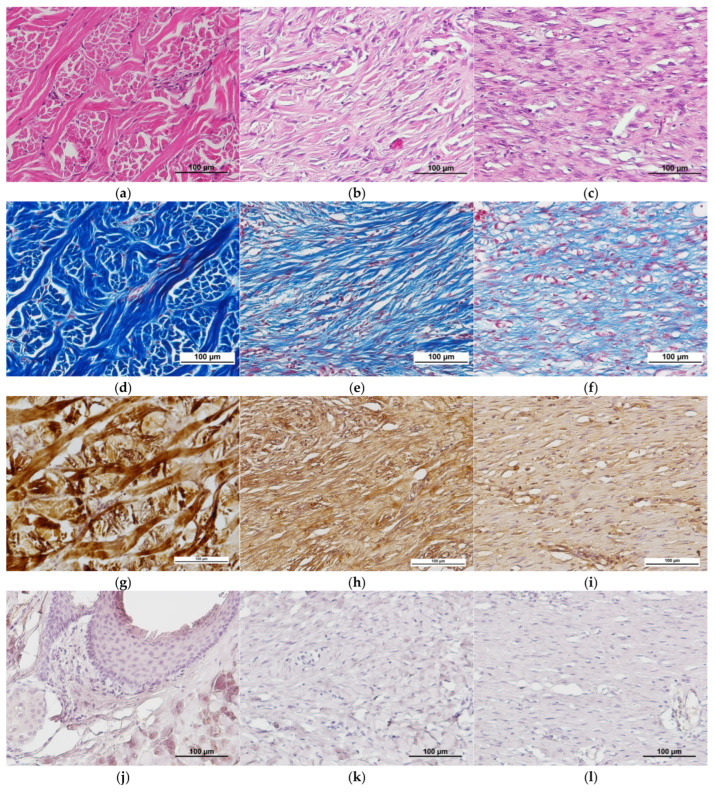

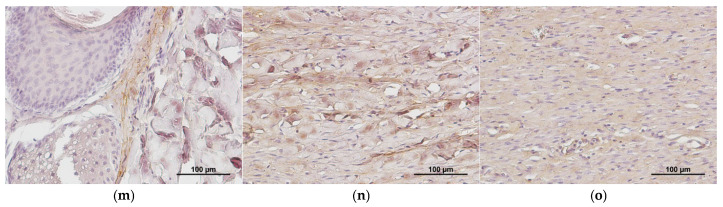
Structure of the graft filling the wound crater: (**a**,**d**,**g**,**j**,**m**) reticular layer of the dermis in the restored skin area; (**b**,**e**,**h**,**k**,**n**) remodeling area; (**c**,**f**,**i**,**l**,**o**) granulation tissue; (**a**–**c**) hematoxylin and eosin staining; (**d**–**f**) Masson staining; (**g**–**i**) staining with Anti-Collagen I antibodies; (**j**–**l**) staining with Anti-Collagen III antibodies; (**m**–**o**) staining with Anti-Collagen IV antibodies.

No significant differences were found during assessment of the condition of tissues in different areas of the wound defects. In the restored dermis, bundles of multidirectional collagen fibers were clearly visible, corresponding to the structure of healthy pig skin (Figure 12a,d). Immunohistochemical staining showed that the main component of the collagen fibers in the extracellular matrix was type I collagen (Figure 12g). Staining for type IV collagen demonstrated that it only occurred in the hair follicles of areas of the restored dermis (Figure 12m). During immunohistochemical examination, type III collagen was not seen in any areas of restored dermis (Figure 12j). According to the literature, type I collagen predominates in pig skin, as in most mammals. The content of type III collagen, which is often associated with type I collagen, in pig skin is rarely discussed, there being only individual studies on this issue [42]. According to the results of Y. Zhang et al. (2020), who studied pig tissues using mass spectrometry, the maximum content of type III collagen in pig dermis samples was 26.54 (±0.35)%. However, the tissues of the animals examined in that study were pre-treated physically, chemically, and biologically, and had an overall sampled collagen content of more than 80%, whereas in native tissues, their content is only up to 51% [43]. It should also be noted that the authors do not provide information about the breed of pigs from which the dermis samples were obtained. The same study provides information about problems in determining type III collagen in the pig dermis using other analytical techniques. Therefore, the lack of detection of any type III collagen in the dermis in our study could be due to a number of factors, such as the small amount of this type of collagen present, the phylogenetic characteristics of pigs, in general, and the specifics of this breed. The efficacy of the antibodies used in our immunohistochemical analysis was also tested on rat skin, where these antibodies clearly stained type III collagen. Thus, the matters of type III collagen content in the pig dermis, the relationship between its level and attribution to a certain breed, and techniques for its determination need to be studied further.

At the border of the restored dermis and maturing granulation tissue of the remodeling area, one could observe partial lysis of the immature matrix and formation of mature multidirectional bundles of collagen fibers (Figure 12b,e). The latter is a natural process of skin structure formation during the late stages of regeneration. Here, type I collagen predominated in the fibers that were forming (Figure 12h), whereas type IV collagen had developed as clusters between the newly forming bundles of mature collagen fibers (Figure 12n). Immunohistochemical examination revealed no type III collagen in the remodeling area, nor in the area of restored skin (Figure 12k). It should be noted that a decrease in fibroblast activity was observed in the remodeling area, although there were still small areas with signs of active collagenogenesis.

Maturing granulation tissue could still be seen in the central parts of the wounds. The extracellular matrix of this maturing granulation tissue was dominated by an amorphous substance, while thin collagen fibers were directed along the wound surface. Fibroblasts with large oval nuclei oriented along the fibers, as well as single leukocytes (eosinophils and/or lymphocytes), were located between the collagen fibers. The granulation tissue contained large thin-walled blood vessels (Figure 12c,f). Types I and IV collagen were found in the extracellular matrix (Figure 12i,o). Note that collagen type I also predominated here compared with the remodeling area. As with other areas, Type III collagen was not detected in the granulation tissue area (Figure 12l).

**Figure 13:**
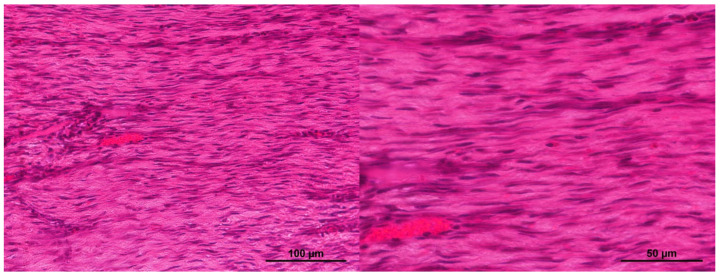
Example of granulation tissue resembling scar tissue in the central areas of control wounds of experimental animals. Hematoxylin–eosin staining.

It should also be noted that, in some cases, in the central areas of the control wounds, the granulation tissue was similar to scar tissue and was formed of thick bundles of horizontally directed collagen fibers, though it also contained a significant number of cells (Figure 13). In such cases, horizontally oriented fibroblasts, each with a narrow, elongated nucleus and poorly developed cytoplasm, were located between the fiber bundles. The blood vessels present were thin-walled and also horizontally oriented.

**Figure 14:**
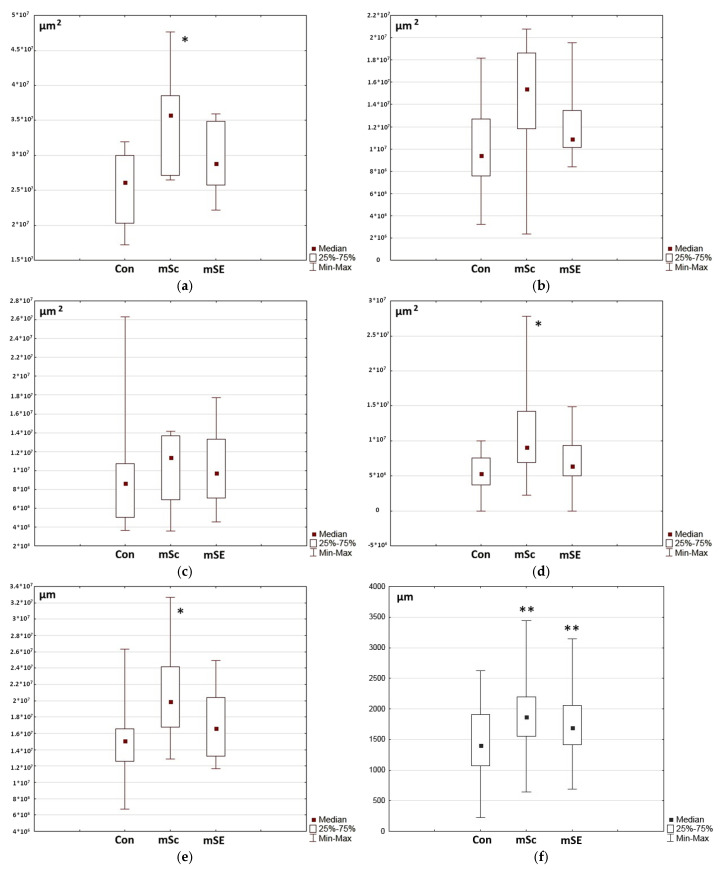
Morphometric studies of the area and thickness of the healing graft zones of wounds on Day 42 of the experimental study. (**a**) Total area of the graft, (**b**) area of restored dermis, (**c**) remodeling area, (**d**) area of the granulation tissue region, (**e**) extent of the granulation tissue and remodeling zones (for a–e *n* = 10 for each type of wound; 1 measurement was taken for each wound defect), (**f**) thickness of the graft in the granulation tissue region (*n* = 50 for each type of wound; 5 measurements having been taken for each wound defect). Note: * *p* ˂ 0.05 ** *p* ˂ 0.001 in comparison with Con, Wilcoxon test.

In order to identify differences in the skin restoration processes in the wound defect areas, a series of morphometric studies were conducted to assess the total areas of the grafts, the areas of the restored dermis, the areas of granulation tissue, and the thickness of the grafts in the central parts of the wounds. Assessment of the total area of the graft revealed that it was statistically significantly larger for wound defects with mSc constructs compared to both the control wounds and wounds with mSEs (Figure 14a). On Day 42 of the study, in wounds with mScs, a significant part of the wound defect was composed of an area with granulation tissue and a remodeling area with active collagen synthesis. This was confirmed by the values obtained during assessments of the extent of the remodeling zone (Figure 14c) and of the granulation tissue zone (Figure 14d), as well as of their total area (Figure 14d). Assessment of the thickness of the graft in the granulation tissue zone also showed significantly higher median values in wounds with mScs (Figure 14f).

There were no statistically significant differences between the groups for this parameter. However, attention should be paid to the scattering values within the samples. They are lowest in wounds treated with mSEs. Similarly, the scatter within the samples in wounds with mSEs is also lower for a number of other analyzed parameters (Figure 14a,b,d–f). This also indicates that the regeneration in wounds with mSEs is more uniform. However, the regeneration process in the mSE wounds was more intensive compared to that for mSc-treated ones, as evidenced by the former’s lower values of granulation tissue space and thickness. The significantly lower values of these parameters in the mSE wounds indicate a higher degree of regeneration completion, which is consistent with the overall histological view of the wound defects on Day 42 of the study (Figure 14d,f).

Summarizing the results, it can be concluded that regeneration in the wound defects studied is not fully completed by Day 42. This is probably due to phylogenetic peculiarities that determine the rate of regeneration in pigs. The results obtained are consistent with the literature data indicating the dependence of healing of pig skin wounds on a range of factors (pig breed, depth and space of the wound, healing conditions, etc.), with the possible duration of this process being up to 6 months [44,45]. At the same time, our analysis of incompletely regenerated wounds allows identification of differences in the activity of the processes involved as well as their specifics. For instance, it was established that, based on the totality of the histological assessment and morphometric studies, mScs and mSEs provided a more intensive regeneration in the area of the wound than occurred in the untreated controls, with the formation of layers and structures similar to healthy tissues. This effect in wound healing is promoted by the biological activity and structure of the biopolymer hydrogel scaffolds of the mSc and mSE grafts, acting to provide niches for cells recruited from healthy surrounding tissues. The scaffold’s capacity to support dynamic reciprocal processes typical of a natural cellular niche has previously been studied in detail in an in vitro model [24]. It was shown that the scaffold provided cellular adhesion for active cell growth and proliferative activity. In turn, the cells cultured in the scaffold changed their microenvironment, secreting growth factors and remodeling their structure, thus changing the properties of the scaffold, in particular the progress of its biodegradation.

In the long term, regeneration processes were more active in wounds with mSEs, as evidenced by the greater maturity of the regenerating tissues compared to those with mScs. However, on Day 7, the opposite had been observed. Apparently, during the initial stages of wound healing, the scaffold’s own biological activity is sufficient to ensure active regeneration, given that it is based on cryoprecipitate, which initially contains sufficient biologically active proteins that can be released into the wound environment to stimulate initial regeneration. Thus, the minor lag in the dynamics of the processes up to Day 7 in wounds with mSEs when compared with mSc grafts may be due to the pre-cultivation of the mSE for three days before implantation. During this period of preliminary cultivation, the ASCs could have changed the scaffold structure, in particular, consuming specific growth factors initially present [24,46]. However, the growth factors in the cell-free scaffold (mSc) soon become depleted as they are consumed during the regeneration. On the contrary, during cultivation, the ASCs in the mSE grafts produce further trophic factors such as VEGF-A, HGF, MCP, SDF-1, IL-6, and IL-8. Such active secretion of trophic factors by ASCs in the SE during cultivation was demonstrated in this study. It has also been established that the ASCs implanted into the wound area as part of the mSE retain their viability for at least 14 days [47]. The accumulated data definitely proves the comprehensive effectiveness of mSEs due to the properties both of the scaffold itself and the prolonged paracrine action of the ASCs, maintaining the regenerative processes over a long period.

## 3. Materials and Methods

### 3.1. Animals

This experimental study was conducted using 8 animals, with 3 further intact animals being used to obtain allogeneic biomaterial. The experimental animals were castrated male pigs aged 6–7 months, hybrids of the Wiesenau breed with the Vietnamese black pot-bellied pig. All animals were quarantined for 1 month in an SPF vivarium. Each was examined for infections and was periodically screened by a veterinarian.

The study protocol was approved by the local ethics committee of the Federal State Budgetary Educational Institution of Higher Education, the “Privolzhsky Research Medical University” of the Ministry of Health of the Russian Federation (protocol No. 5 dated 10 March 2021). All procedures involving animals were carried out in the operating room of the vivarium in compliance with aseptic and antiseptic regulations and the requirements of the European Convention for the Protection of Vertebrate Animals Used for Experimental and Other Scientific Purposes (No. 123 of 18 March 1986; ETS No. 170, 22 June 1998, Strasbourg).

### 3.2. Animal Sedation and Anesthetization

Surgical interventions, dressings, and sample collection were performed using combined anesthesia. Animal induction was conducted by intramuscular administration of Zoletil 100 (Virbac, Carros, France)—6 mg/kg and Xyla 2% (Interchemie werken “De Adelaar”, Limburg, Netherlands)—0.3 mg/kg. After induction, tracheal intubation was performed with subsequent synchronized intermittent mandatory ventilation of the lungs (SIMV). During the intervention, the animal’s respiration, hemodynamics, and body temperature were monitored, while anesthesia was maintained by intermittent administration of Propofol-lipuro (B.Braun Melsungen AG, Melsungen, Germany)—5 mg/kg and Zoletil 100—2 mg/kg.

### 3.3. Preparatory Procedures

**Figure 15:**
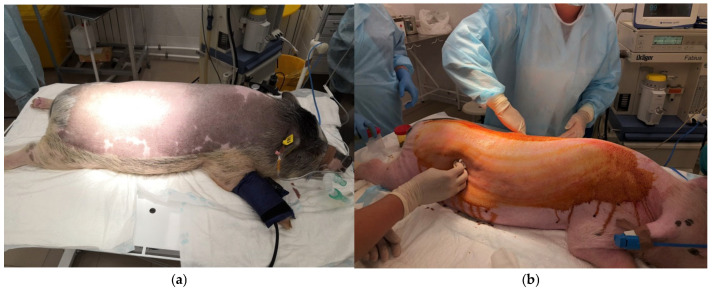
Preparation of the animal for surgical intervention: (**a**) anesthetization and intubation of the animal under monitoring; (**b**) treatment of the surgical area before surgery.

The animals were cleaned using a shower with a body sponge and antiseptic soap. Then, the hair in the areas of the intended surgical intervention was carefully shaved using an animal clipper. The animal was transported to the operating table on a gurney. The surgical area was first treated with a 5% iodine solution in alcohol, then with 70% ethyl alcohol. All further surgical procedures were performed by an experienced surgeon (Figure 15a,b).

### 3.4. Monitoring of Animals During Surgery

The animals were monitored by professional anesthetists. During the surgery, blood pressure, heart rate, blood oxygen saturation, respiration, and ventilation were monitored using a stationary device and a veterinary monitor (Mindray uMEC12 Vet Advance, Mindray, Shenzhen, Guangdong, China; Mindray Veta 5, Mindray, Shenzhen, Guangdong, China).

### 3.5. Extraction of Biomaterial to Obtain Pig Mesenchymal Stem Cells and Pig Blood Plasma

**Figure 16:**
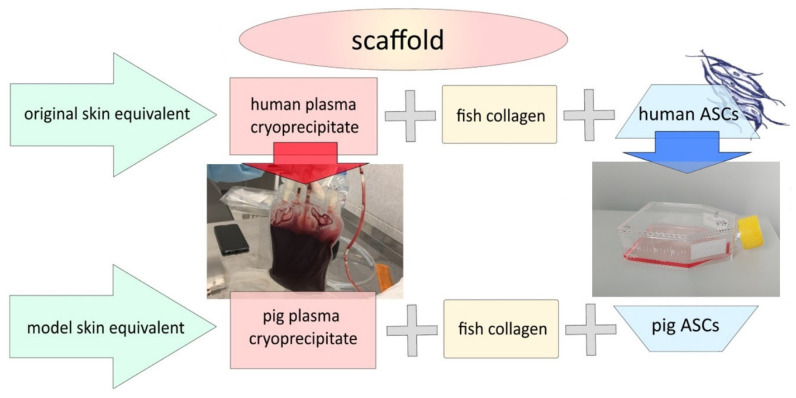
Creating a model skin equivalent for studies on pigs.

To conduct this experimental study on the animals, a model skin-equivalent (mSE) was needed—a method corresponding to the “homologous drug” strategy. To form such an mSE, the original allogeneic components—human adipose-derived stem cells (hASCs) and human blood plasma cryoprecipitate in the source skin-equivalent intended for use in humans—were replaced by allogeneic ones for pigs (pig adipose-derived stem cells—pASCs and pig blood cryoprecipitate) (Figure 16).

Three intact animals were used as sources of pASCs and pig blood plasma. To obtain adipose mesenchymal stromal cells, subcutaneous fat was collected from each animal’s back in a volume of 10–30 cm^3^. Sampling was performed using an ACCULAN^®^ 3Ti Dermatome GA 670 electrodermatome (B.Braun, Tuttlingen, Germany). The electrodermatome was used to sequentially remove a layer of skin and the upper layer of subcutaneous fat (1.2 mm) from the surgical area. Then, specimens of the subcutaneous fat were collected (flap thickness, 1.2 mm, flap width up to 10 mm; layer depth up to 2.4 mm). To remove blood impurities, the samples were repeatedly and thoroughly washed in Hank’s solution with antibiotics—100 U/mL penicillin, 100 μg/mL streptomycin (PavEko LLC, Moscow, Russia) and placed in vials with transport medium.

To obtain blood plasma, blood was taken from the renal vein. The blood was collected in RAVIMED containers (Ravimed Sp. z o.o., Legionowo, Poland) containing CPDA-1^®^ hemopreservative solution.

### 3.6. Obtaining an ASC Culture

The subcutaneous adipose tissue extracted in the operating room of the vivarium was placed in a vial with transport medium and delivered to the biotechnology laboratory of the University Clinic of the Privolzhsky Research Medical University. Subsequent work was carried out in the sterile conditions of a clean room using Class II laminar flow hoods with vertical flow (Kojair Tech Oy, Vilppula, Finland).

The stromal–vascular fraction of the adipose tissue was isolated by heated enzymatic treatment using collagenase type I (Stemcell Technologies, Vancouver, BC, Canada). The resulting cell suspension was seeded into culture flasks (Corning, New York, NY, USA) for further cultivation. The culture was grown in a CO_2_ incubator (5% CO_2_, +37 °C, absolute humidity) with the medium changed twice a week. The complete growth medium had the following composition: DMEM F-12 (Gibco™, Thermo Fisher, Waltham, MA, USA), 10% fetal bovine serum (FBS) (Gibco™, Thermo Fisher, Waltham, MA, USA), 2% glutamine, antibiotics (penicillin/streptomycin; LLC PanEco, Moscow, Russia). A 0.25% trypsin solution in Versene (Gibco™, Thermo Fisher, Waltham, MA, USA) was used for subculturing. The cell culture was re-seeded when a subconfluent monolayer had been formed (70–80%). Cultures of 3–4 passages were used in the study. Before use in the experiment, the cell cultures were checked for sterility.

The immunophenotype of the pASC cells was determined by multicolor analysis using a direct immunofluorescence reaction. A panel of monoclonal antibodies CD 44 FITC, CD 90 PerCP-Cy5.5, CD 10 PC7, CD 45 PE (Becton Dickinson, Franklin Lakes, NJ, USA) with the corresponding isotype controls on a FACS CANTO II cytofluorimeter (Becton Dickinson, USA) was used. Stained cells were incubated for 30 min and washed for further determination of the immunophenotype. According to the phenotyping results, more than 90% of the cells had characteristics typical of MSCs: CD90+, CD 44+, CD 10+, CD 45−.

The viability of the cells before introduction into the composite for mSE formation was 98–99%.

### 3.7. Formation of the Model Scaffold Carrier and Skin Equivalents

To form the model biopolymer hydrogel, cell-free scaffold (mSc) and the mSE derived from it, a composite based on blood plasma cryoprecipitate was used in line with a previously described technique [48]. The composite for forming the mScs and mSEs contained PEGylated proteins of pig plasma cryoprecipitate and type I collagen (cod type I collagen isolated from cod skin) [49]. When forming the mSEs, a suspension of pASCs in a phosphate buffer solution (PBS) was introduced into the composite. The concentration of pASC cells was 1.2 × 10^5^ per 1 mL of the composite. To create the cell-free scaffold model, a volume of PBS was introduced into the composite equal to that of the pASC suspension. To polymerize the composite, it was added with a thrombin–calcium mixture: 80 IU/mL thrombin (RPA ‘RENAM’, Moscow, Russia) in 1% CaCl_2_ solution. The resulting mScs and mSEs were cultured for 3 days in complete growth medium in a CO_2_ incubator (+37 °C; CO_2_ 5%; absolute humidity). Before transplantation into animals, the mSc and mSE samples were washed three times with phosphate buffer solution.

It should be noted that the absence of cytotoxicity of the cell-free mSE scaffold had been shown during earlier developments of the model skin equivalents (mSEs). It was also confirmed that the mSE scaffold provides conditions for the placement and three-dimensional growth of cells similar to those of the SE scaffold. Cellular events that occurred during the cultivation of the mSE and SEs were also comparable [50].

### 3.8. Wound Formation

**Figure 17:**
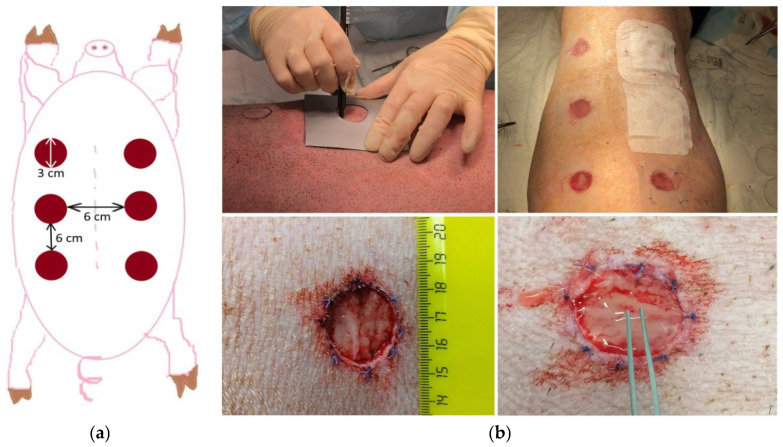
Wound formation in animals. (**a**) Layout of wound locations in experimental animals, (**b**) formation of wounds and grafting of mScs/mSEs.

The areas of wound defect formation were marked along the animals’ spines using a tracer (Figure 17a). Six full-thickness degloving wounds (three on the right and three on the left), 3 cm in diameter and down to the level of the subcutaneous fat (panniculus adiposus), were formed at a distance of at least 6 cm from each other on both sides of each animal’s spine (Figure 17b). As a result, each animal provided for 2 wounds of three types: 2 control (Con) to be left without treatment; 2 experimental wounds (mSEs) for grafting of model skin equivalents (scaffold with encapsulated cells—ASCs) into the wound area; and 2 experimental wounds (mScs) for grafting of the model cell-free scaffold into the wound area. The wound edges were marked by suturing with non-absorbable suture material.

All wounds were covered with a siliconized protective coating (Transparent Dressing, Winner Medical Co., Ltd., Shenzhen, China) and a fluid-retaining dressing (Cosmopor E, Paul Hartmann AG, Heidenheim an der Brenz, Germany) (Figure 17b). To reduce the risk of accidental loss of the dressing and further infection of the wounds, additional protective dressings were used.

During the entire observation period, the wounds were moistened twice a day with a physiological solution containing gentamicin (0.022 mg/mL).

### 3.9. Post-Surgery Period

After the surgical intervention, test animals were brought out of anesthesia with a caffeine injection and placed individually in a special stall, which partially limited the animal’s movement in order to avoid accidental loss of dressings and potential infection of the wounds.

From 4 to 6 h after surgery, pain was relieved by administering an analgesic (Ketoprofen 2–3 mg/kg) intramuscularly every 12 h, if needed. During the first 24 h after surgery, careful monitoring of each animal’s cardiovascular and respiratory functions, temperature, and the presence of wound bleeding was conducted.

Throughout the entire post-surgery period, the general clinical condition of the animals (behavior, activity level, food and water consumption, body temperature) was monitored on a daily basis. Weekly, the animals were re-bandaged, involving a complete change in dressings, with a full clinical examination being performed at the same time (the condition of the skin and mucous membranes was specifically checked).

### 3.10. Animal Euthanasia

Animals were euthanized after predetermined test periods (3 animals were sacrificed on Day 7, and 5 animals were sacrificed on Day 42) by means of narcotization. The time of death was recorded by an anesthetist. During euthanization, the following was assessed: the general condition of the animals, the appearance of the wounds and surrounding tissues, the color of the tissue, signs of inflammation, and any visual presence of the graft elements. After the animal was sacrificed, tissues were sampled from the wound areas. Each stage of the procedure for biomaterial sampling was recorded photographically. During this surgery, the wound area was excised for morphological examination. The isolated tissues were transferred for further processing to an experienced histologist, who conducted a blind examination of the wound area tissues.

### 3.11. Morphological Studies of Biomaterial

#### 3.11.1. Light Microscopy

Tissue samples from the wound defect area were fixed in 10% neutral formalin solution, then dehydrated in an alcohol bath and embedded in paraffin. The paraffin blocks were cut into 5 μm thick sections using a Leica SM 2000R microtome (Leica Macrosystems, Wetzlar, Germany). The sections were dewaxed, stained with hematoxylin and eosin (Biovitrum, Moscow, Russia) and Masson (Biovitrum, Moscow, Russia), dehydrated, and mounted under coverslips in synthetic Vitrogel medium (Biovitrum, Moscow, Russia). The material was photo-recorded using both a BLM microscope (LOMO, St. Petersburg, Russia) equipped with an MS-20 digital camera (LOMO, St. Petersburg, Russia) with the MCview software (LOMO, St. Petersburg, Russia), and with a Nikon Eclipse 80i microscope with a Nikon Ds-Fi1 camera (Nikon, Tokyo, Japan). Morphometric processing was conducted using a BLM microscope (LOMO, St. Petersburg, Russia) equipped with an MS-20 digital camera (LOMO, St. Petersburg, Russia) with the MC View software.

#### 3.11.2. Immunohistochemistry

The following antibodies were used to assess the neoangiogenesis and the proliferation intensity in the wound area: Anti-CD31 (ab182981, Abcam, Cambridge, UK) at a concentration of 1:100 and Anti-Ki67 (ab182981, Abcam, Cambridge, UK) at a concentration of 1:1000. The collagen content in the intercellular matrix was determined using Anti-Collagen I, Anti-Collagen III, and Anti-Collagen IV antibodies (ab34710, ab23445, and ab6586 (Abcam, Cambridge, UK)) at concentrations of 1:200, 1:200, and 1:400, respectively. All processing steps were conducted in accordance with the recommendations of the antibody manufacturer.

#### 3.11.3. Morphometric Examinations

A BLM microscope equipped with an MS-20 camera with MC View software (LOMO, St. Petersburg, Russia) was used for morphometric processing. Quantitative assessment of the number of positively stained cells and of the percentage of positively stained areas in the total area was carried out over at least 10 fields of view at a magnification of 40×.

### 3.12. Statistical Analysis

The results were processed using nonparametric statistics involving the Mann–Whitney U-test and a Wilcoxon’s paired-comparison test (STATISTICA 6.0 software package).

## 4. Conclusions

The investigations we conducted have demonstrated that the biopolymer hydrogel scaffold used became integrated into the recipient tissues during the early stages of regeneration, ensuring both the recruitment of cells and the maintenance of their proliferative activity, as well as accelerated maturation of the granulation tissue. It was found that one of the mechanisms for regeneration following scaffold implantation was the recruitment of eosinophils to the wound area, resulting in a cascade of releases of biologically active substances during degranulation and their ability to participate in the regulation of regeneration. In the later stages, these activities resulted in the formation of tissues that completely filled the crater of the wound bed and were comparable to healthy tissues in both thickness and structure. Encapsulation of ASCs within the scaffold increased its effectiveness, including faster remodeling of the graft connective tissue and acceleration of regeneration. Both the cell-free scaffold and the skin equivalent, with ASCs attached to such a scaffold, prevented the formation of scar tissue in the wound area. Therefore, this biopolymer hydrogel scaffold provides an effective tool to be used in regenerative medicine for the restoration of skin wounds of various origins.

## Figures and Tables

**Table 1:** Skin equivalents used in clinical practice.

Brand	Manufacturer	Product Description
Integra^®^ DRT (Dermal Regeneration Template) [5,6]	Integra LifeSciences (Princeton, NJ, USA)	Thin, cell-free silicone film covering a porous matrix made of bovine collagen and glycosaminoglycans
Apligraf^®^/Graftskin^®^ [6,7]	Organogenisis (Canton, MA, USA)	Fibroblasts and collagen combined in a dermal matrix seeded with keratinocytes to form an epidermal-like layer
Epicel^®^ [8]	Genzyme (Cambridge, MA, USA)	Autologous keratinocytes grown ex vivo in the presence of a mouse fibroblast feeder layer
TransCyte^®^/Dermagraft^®^ Advanced Tissue [6,9]	Shire Regenerative Medicine (Lexington, MA, USA)	Cryopreserved skin substitute: human fibroblasts seeded onto a polymer mesh and cultured ex vivo
TransCyte^®^ [6,10]	Shire Regenerative Medicine (Lexington, MA, USA)	Human allogeneic fibroblasts derived from newborn foreskin seeded onto a silicone-coated degradable nylon mesh sponge and cultured ex vivo for 4–6 weeks; these secrete extracellular matrix components and growth factors
OrCel^®^ [6,11]	FortiCell Bioscience (New York, NY, USA)	Human epidermal keratinocytes and dermal fibroblasts cultured in separate layers with type I bovine collagen
Alloderm^®^ [12]	Strattice^®^ LifeCell Co.^®^ (Branchburg, NJ, USA)	Cell-free matrix of cadaver skin
Laserskin^®^ [10]	Fidia Advanced Biopolymers (Turin, Italy)	Autologous keratinocytes and fibroblasts from skin biopsy samples, cultured on a laser-microperforated biodegradable matrix of benzyl-esterified hyaluronic acid
PermaDerm^®^ [10]	Regenicin, Inc. (New York, NY, USA)	Autologous keratinocytes and fibroblasts cultured on a bovine collagen support
StrataGraft^®^ [6,10]	The Luminis Group, Ltd. for Stratatech Corp (Madison, WI, USA)	Patented, immortalized keratinocytes of the NIKS^®^ (Normal Immortal Keratinocytes) line, together with dermal fibroblasts on a collagen support
GraftJacket^®^ [10]	Wright Medical Technology (Arlington, TN, USA)	Micronized decellularized human dermis with dermal matrix and preserved basement membrane for ingrowth of blood vessels
Biobrane [6]	Smith & Nephew (London, UK)	Biosynthetic adhesive wound dressing constructed with an outer silicone membrane and an inner nylon mesh with added type I pig collagen
EpiDex^®^ [13]	Anika Therapeutics (Bedford, MA, USA)	An epidermal equivalent derived from isolated hair follicle keratinocytes cultured on a matrix
BioSeed^®^ [13]	BioTissue Technologies AG (Freiburg im Breisgau, Germany)	An epidermal equivalent derived from autologous keratinocytes and a fibrin sealant

## Data Availability

The original contributions presented in this study are included in the article. Further inquiries can be directed to the corresponding author(s).
